# The Impact of Carbonic Maceration Pretreatment on the Convective Drying of Seedless Grapes: RSM Optimization, Drying Characteristics and Microstructure

**DOI:** 10.1002/fsn3.70066

**Published:** 2025-03-08

**Authors:** Nursaç Akyol Kuyucuoğlu, Muhammed Mustafa Özçelik, Merve Seçil Bardakçı, Erdoğan Küçüköner, Erkan Karacabey

**Affiliations:** ^1^ Department of Food Processing Bingol Vocational School of Food, Agriculture and Livestock, Bingol University Bingol Turkey; ^2^ Department of Food Engineering Faculty of Engineering and Natural Sciences, Suleyman Demirel University Isparta Turkey

**Keywords:** Drying Characteristics, Pretreatment, Response Surface Methodology, *Vitis vinifera* L.

## Abstract

In the last 2 decades, Turkey and the United States have produced half of the world's dried grape production. Sultani seedless grape, used as a material in the study, is the most important grape variety since being economically growing in Turkiye for dried grape production and its exportation. The aim of the present study was to investigate the drying of seedless grapes by carbonic maceration (CM) pretreatment and following hot air drying. By the way statistical analysis was carried to determine how the process parameters affected the drying behavior of grape samples. Finally, the studied parameters of both process steps, pretreatment and following drying, were simultaneously optimized according to the investigated responses. The results showed that drying temperature and CM process parameters significantly affected the drying efficiency. Notably, when compared to the control, the CM treatment accelerated the drying process by more than 24%. Furthermore, antioxidant potential, surface color, and texture were found to be better preserved in the dried grapes treated with CM. While pH, hue, TPC, and TEAC values of optimum dried grapes were significantly higher, TA and HMF values were remarkably lower than control samples (without CM pretreatment) (*p* ≤ 0.05). Throughout CM pretreatment and following the drying process, the dependent variables, being elasticity, antioxidant capacity, drying time, and hue value, were optimized by the response surface methodology (RSM). The optimal drying temperature (DryT), CM pressure (CMP), CM temperature (CMT), and CM time (CMt) parameters were as 77°C, 0.30 MPa, 4°C, and 8 h, respectively. For drying operations, CM's significant advantages should be considered in terms of enhancing the process and the product. As a result, this study offers significant findings in this context. The CM has been found to be an effective technique utilized before drying grapes based on the results that were achieved.

## Graphical abstract

Carbonic maceration (CM) is an effective technique as a pretreatment of the drying process. In particular, the CM treatment accelerated the drying process of grapes by more than 24% compared with the control. For drying operations, CM's significant advantages should be considered in terms of enhancing the process and the product. Additionally, antioxidant activity, surface color, and texture were better preserved in dried grapes treated with CM.

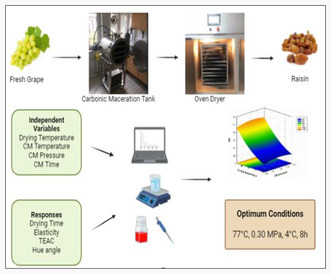

## 1 Introduction

Grape ( *Vitis vinifera* L.) is a significant agricultural crop around the world. It is consumed in its raw form, dried, and/or processed, especially into wine, depending on the variety and demand. Every year, an estimated 75 million tons of grapes are produced. About half of the grapes are used in the wine fermentation process all over the world, while the rest are consumed fresh or dried (FAO and OIV 2016). Sultani is the well‐known grape variety and the most popular seedless grape cultivar in Turkey and all other grape‐growing countries (Altindisli and Ozsemerci 2013; Kupe et al. 2021; Pawar et al. 2023) especially for drying. Fresh grapes have a high moisture content (80%–85% w.b.). Even if stored under refrigerator conditions, they are perishable (Giuseppina Adiletta et al. 2015). Therefore, as mentioned above, a certain amount is processed, and one of the methods for grape processing is drying. Drying fruits and vegetables with high water content, as well as being heat‐sensitive, sugary, sticky, and containing peel and seeds, using traditional methods is quite challenging (Ozcelik et al. 2023). The grape surface is covered in a waxy cuticle layer that limits gas exchange while also shielding the fruit from diseases and physical harm (Esmaiili et al. 2007; Pawar et al. 2023; Srivastava et al. 2021). Therefore, the drying process of grapes is more difficult than others (Bai et al. 2013). To get rid of the waxy covering, various pretreatments (chemical or physical) are used before drying the grapes (Adiletta et al. 2016; Giuseppina Adiletta et al. 2015; Bai et al. 2013; Carranza‐Concha et al. 2012; Miraei Ashtiani et al. 2020; Pahlavanzadeh et al. 2007; Pawar et al. 2023). In chemical pretreatments, the products are exposed to chemicals for a specific period and at a controlled temperature to increase water permeability by weakening the wax coating on the surface of the grapes (Srivastava et al. 2021). However, chemical pretreatments cause soil and ecosystem‐damaging wastewater and result in residue on the product (Adiletta et al. 2016; Bai et al. 2013; Pawar et al. 2023; Wang et al. 2017; Wang et al. 2016). Thus, alternatives to replace chemical ones have been sought for a while. Carbonic maceration (CM) pretreatment is one of the alternatives. It has been proven not to show any adverse effects on the sensory and nutritional properties of the product during the drying process, and its effect has been investigated as an environmentally friendly method (An et al. 2020; An et al. 2018; Liu et al. 2014; Ozcelik et al. 2022; Turgut et al. 2018b; Wang et al. 2014; Zhao et al. 2016). CM, which Michael Flanzy discovered in 1934, has been used to improve the flavor of grape wines (Flanzy et al. 1987). Recently, the CM method has been preferred as a pretreatment before the drying process and has been determined to be promising (Liu et al. 2014; Wang et al. 2014). Carbon dioxide application is a nonthermal process. While carbon dioxide application prevents the growth of microorganisms at ambient pressure, it can diffuse some microorganisms at higher pressures. Carbon dioxide provides a cytoplasmic pH drop in the cell, thanks to its enhanced dissolution power. Thus, key enzymes become ineffective, and the extraction of intracellular substances gets easier (Gunes et al. 2005). On the other hand, with the pretreatment of CM, the cell wall collapses, and its permeability is manipulated in favor of drying. The bound water in the structure turns into free form in the plant tissue. In this way, capillary spaces increase and vacuoles distance. Also, while intracellular pH decreases due to the pressure effect, free water content, and phenolic substance extraction increase (An et al. 2020; Liu et al. 2014; Ozcelik et al. 2022). In the meantime, polyphenolic loss can be reduced by the inactivation of polyphenol oxidase and peroxidase enzymes with CM treatment (An et al. 2018). Since the CM technique increases cell permeability, it reduces the drying time of the product. Therefore, quality losses that may occur in the product can be minimized. In this context, the CM technique is hopeful, especially for drying fruits with a wax layer such as grapes. There are few studies in the literature that feature strawberries (Ozcelik et al. 2022), Chinese ginger (An et al. 2020), plums (An et al. 2018), tomatoes (Turgut et al. 2018a), potatoes (Zhao et al. 2016), red grapes (Wang et al. 2014), and pepper (Liu et al. 2014) dried with the CM treatment technique.

Response surface methodology (RSM) is a mathematical and statistical approach for efficient experimental design and is widely used for reducing the number of experiments, factors, and their levels (Chelladurai et al. 2021). Also, it is an effective method for modeling and optimizing several variables and their interactions on one or more response variables (Ozcelik et al. 2022). The Box–Behnken design (BBD) is a simpler and more effective type of RSM than other three‐level factorial designs in which independent variables are tested at three levels to create the response model and fit the output (expected response) into a quadratic model (LaPanse et al. 2021; Martin‐Garcia et al. 2020).

CM pretreated drying optimization with convective drying using RSM is very scarce in the literature (Ozcelik et al. 2022; Serhat Turgut et al. 2018), and the optimization of the CM pretreatment and following drying conditions for drying grapes has not been in the literature. This gap in literature has been figured out by Pawar et al. (2023) as well, where they mentioned the promising outcomes of CM as a pretreatment application before drying grape samples and declared that the large‐scale application of carbonic maceration needs to be explored.

The aim of this study was to dry seedless grapes of the Sultani variety by applying CM pretreatment. CM conditions (pressure, time, and temperature) and drying temperature (DryT) were optimized using the RSM to reduce process time and maintain quality. The study examined the impact of pretreatment and drying on grape drying time, texture, color, and bioactive compounds. Additionally, under the determined conditions, the samples were dried with or without pretreatment under the optimum drying conditions. The changes caused by CM pretreatment on the final product were examined.

## 2 Material and Methods

### 2.1 Material

The Sultani seedless grape ( *Vitis vinifera* L.) used in this study was supplied by a local producer in Alaşehir, Manisa, the mainland of the target variety, in Turkey. Grapes harvested at similar maturity were kept in a fridge between 4°C–6°C until the process. Before processing, the grapes were cleaned again, washed, and excess water was removed.

### 2.2 Experimental Design and Optimization

In order to maximize the benefits of a system, process, or product, optimization is a way of making it operate efficiently. The phrase “optimization” has been widely used to refer to the procedure to identify the optimal response by applying a method (Araujo and Brereton 1996). Traditional optimization methods are based on monitoring the influence of one factor at a time on an experimental response. In this method, one parameter is changed and its effect on the target response is visualized, whereas the remaining ones are kept constant. This optimization method is known as one‐variable‐at‐a‐time. The main disadvantage of this method is not considering the potential interaction between studied variables. Thus, some potential effects are not examined (Lundstedt et al. 1998). Additionally, in that method, the number of trials is too high to conduct all of them. Thus, it is time‐consuming, high cost, more labor requirements, and high consumption of reagents and materials. In order to overcome those disadvantages, using multivariate statistical techniques is suggested as an optimization of analytical procedures. Among the most relevant multivariate techniques used in analytical optimization is response surface methodology (RSM). Thus, in this research, the RSM BBD was used for the optimization and modeling of the drying and CM pretreatment conditions. For the experiment, time, pressure, temperature of the CM pretreatment, and the DT were identified as independent variables (Table 1). Based on the preliminary test results, the minimum (Mnv) and maximum (Mxv) factor levels of the independent variables were determined and consisted of 27 experiments (Table 2).

**TABLE 1:** Defining independent variables of BBD.

Independent variables	Mnv	Mxv
X_1_: DryT (°C)	60	80
X_2_: CMP (MPa)	0.1	0.3
X_3_: CMT (°C)	4	40
X_4_: CMt (h)	8	24

**TABLE 2:** Experiment design for CM pretreatment and convection drying process conditions optimization.

Run number	Block number	CMP (MPa)	CMT (°C)	CMt (h)	DryT (°C)
1	2	0.3	22	16	60
2	2	0.3	22	16	80
3	2	0.2	4	8	70
4	2	0.2	4	24	70
5	2	0.2	40	8	70
6	2	0.1	22	16	60
7	2	0.2	22	16	70
8	2	0.1	22	16	80
9	2	0.2	40	24	70
10	3	0.3	22	8	70
11	3	0.1	22	8	70
12	3	0.3	22	24	70
13	3	0.2	4	16	60
14	3	0.1	22	24	70
15	3	0.2	40	16	80
16	3	0.2	4	16	80
17	3	0.2	22	16	70
18	3	0.2	40	16	60
19	1	0.2	22	16	70
20	1	0.3	40	16	70
21	1	0.1	40	16	70
22	1	0.2	22	8	60
23	1	0.2	22	8	80
24	1	0.3	4	16	70
25	1	0.1	4	16	70
26	1	0.2	22	24	80
27	1	0.2	22	24	60

Minitab Statistical Software was used for RSM (Minitab Inc., State College, USA) and model adequacy and regression tests were evaluated by *R* ^2^ and lack‐of‐fit value. All runs were randomized, and six experimental responses (dependent variables) were presented (moisture, water activity, drying time, elasticity, total antioxidant capacity [TEAC] and hue values). The experimental data were used to fit a second‐order polynomial model in order to determine the regression coefficients (*β*). RSM analysis employed a generalized second‐order polynomial model, represented by Equation (1).

(1) $$Z=\beta_{0}+\sum_{i=1}^{3}\beta_{i}X_{i}+\sum_{i=1}^{3}\beta_{\mathrm{ii}}X_{i}^{2}+\sum_{i=1}^{2}\sum_{j=i+1}^{3}\beta_{\mathrm{ij}}X_{i}X_{j}$$

*Z* is the dependent variable, *X* is the independent variable, and the constant coefficient is defined as *β*_0_ for intercept, *β*_*i*_ for linear, *β*_*ii*_ for quadratic, and *β*_*ij*_ for two factors interaction coefficient.

### 2.3 Pretreatments

Approximately 2500 g of grapes were processed for each trial of the experimental design. By virtue of the pressure tank operating for approximately half an hour just before treatment, the temperature was achieved to attain the desired values. Grapes were placed in small clusters on stainless steel trays in a 750 mm length tank with a diameter of 500 mm. The lid of the tank was closed tightly to prevent leakage. The oxygen was removed from the vacuum inlet of the tank by a vacuum pump. CO_2_ gas was allowed to fill up the CM tank until the internal pressure reached the specified level. When the internal pressure attained the desired values, the time measurement was started for CM. After CM pretreatment, the samples were taken from the tank, placed on a drying tray, and the following step, drying started.

### 2.4 Drying Process

The grapes were dried in a standard hot air drier controlled by a PLC (EKSIS LTD., TK‐LAB Model, Isparta/Turkey). The drying temperature was at the specified value in the design table, the constant air flow velocity was 2 m/s, and the tray rotational speed was 15 rpm. According to the literature, a moisture level < 20% was the desired level for raisin (Krokida et al. 2003). Therefore, in this study, the same moisture level was followed, and when it was attained, the drying was stopped. The change in moisture content was tracked by electronically weighing the sample during drying. Dried samples were stored in a laboratory‐type deep freezer at −45°C in a polyethylene vacuum packaging until analysis. For the remaining analysis, besides the raisin samples obtained according to the process defined at the experimental design table, the following samples were also achieved to handle for comparison (Table 3).

**TABLE 3:** Sample codes and specifications of fresh, CM treated, and control samples.

Sample	Sample code	CM pretreatment at its optimal conditions	Drying at its optimal conditions
Fresh	C101	✗	✗
Raisin @ optimal condition	C102	✓	✓
Raisin @ optimal convectional drying	C103	✗	✓

### 2.5 Moisture Content (%), Water Activity (a_w_), and Soluble Dry Matter (°Bx)

To determine the moisture content (MC) of fresh and dried grapes, a moisture analyzer was used (DBS 60–3, Kern & Sohn GmbH, Germany). For each test, approximately 6–7 g of sample was used, and the moisture content was measured at 105°C ± 1°C for 24 h (Czaplicka et al. 2022; Kiliçkan et al. 2010).

The water activity (aw) of samples was assessed at ambient temperature through the utilization of a water activity analyzer (Thermoconstanter TH 200, Novasina, Axair Ltd., Switzerland). Brix (°Bx) measurement was carried out using a refractometer (HI 96801, Hanna, Germany) at 25°C.

### 2.6 Titratable Acidity and pH Determination

The pH value of the samples prepared by homogenization of dried grapes was measured potentiometrically using a pH meter (HI 2211, Hanna, Romania). The titration acidity value of the samples (%) was expressed as tartaric acid equivalent.

### 2.7 Color Measurement

To determine the color of samples, a colorimeter (NH310 Colorimeter, Shenzhen 3NH Technology LTD, China) was utilized. BI and ΔE values were calculated based on the previously published study (Aydin et al. 2023).

### 2.8 Hydroxymethylfurfural Content

Hydroxymethylfurfural (HMF) analysis for the dried grape samples was carried out by modifying the method suggested by Hidalgo and Pompei (2000). For this purpose, 5 mL of ultrapure water was added to 2.5 g of dried grape and homogenized. Then the sample was vortexed for 120 s and centrifuged at 2594 *g* for 10 min. The supernatant was prepared for HPLC injection by passing it through a 0.45 μm filter. HPLC conditions used were reported by Turgut et al. (2018b). The liquid chromatography system (1260 Infinity series, Agilent, Palo Alto, CA, USA) equipped with an autosampler and photodiode array detector, was used for HMF detection at 282 nm. 10 μL of the sample was injected onto a reversed‐phase C18 column (ACE, 5 μm, 250 mm × 4.6 mm, Avantor, Aberdeen, Scotland). An isocratic solvent consisting of a 1:10 ratio of methanol to ultrapure water was employed at a flow rate of 0.5 mL/min. The column was maintained at 30°C, and HMF concentration was determined using a standard curve. Findings were presented as mg HMF/g dry weight.

### 2.9 Extraction of Bioactive Compounds

The extract from the dried grape samples was obtained by adapting the method that Toor and Savage (2006), with some modifications. In summary, 10 mL of a blend consisting of acetone, water, and acetic acid in a ratio of 70:29.5:0.5 (v/v/v) was introduced to 4 g of desiccated grapes and then ground with a pestle. The crushed mixture was placed in centrifuge tubes, then vortexed for 1 min and sonicated for 5 min. The sonicated sample was vortexed for another 5 min and centrifuged at 2594 *g* for 10 min. The procedure was repeated twice, and the supernatants were combined. The supernatant was taken into the balloon and evaporated for 20 min in an evaporator (Hei‐VAP precision rotary evaporator, Heidolph, Germany) at 40°C.

### 2.10 Total Phenolic Content

Total phenolic content (TPC) of grape sample was determined using the Singleton and Rossi (1965) method. The absorbance was measured at 760 nm using a UV/VIS spectrophotometer (T70, PG Instruments, UK). The findings were represented as “mg gallic acid equivalent (GAE) per gram of dry substance”.

### 2.11 Determination of Antioxidant Activity

To determine the Trolox equivalent antioxidant capacity (TEAC) of samples, the ABTS method reported by Re et al. (1999) was used after modification. For this purpose, 7 mM ABTS solution that contained 2.45 mM K_2_S_2_O_8_ was prepared for the analysis and maintained for 12–16 h in the dark, forming an ABTS + radical. To achieve the absorbance of 0.700 ± 0.02 at 734 nm, ABTS solution was diluted with pure ethanol (%99). After that 10 μL of the extract was combined with 990 μL of prepared ABTS solution (0.700 abs). Two consecutive measurements were carried and recorded at 0 and 6 min of reaction. The results were calculated according to the Equation (2) and given as “mg TE/g dry matter.”

(2) $$\%\text{Inhibition}=\;\left[\frac{\begin{array}{ccc} {\mathrm{Ab}}_{0} & — & {\mathrm{Ab}}_{1} \end{array}}{{\mathrm{Ab}}_{0}}\right]\times 100$$

where, Ab_0_ is the initial absorbance value of the mixture and Ab_1_ is the absorbance value of the mixture taken after 6 min of reaction.

### 2.12 Determination of Total Flavonoid Content

The determination of total flavonoid content (TFC) in the samples was conducted using the procedure published by Toor and Savage (2006). Approximately 10 g of sample was treated with 15 mL 96% (v:v) ethanol. The sample collected on the filter paper was extracted with 140 mL of 96% ethanol in a Soxhlet extractor for 1 h. 5 mL of the extract was taken and 0.3 mL of 5% (w/v) sodium nitrite (NaNO_2_) was added. The sample was incubated in dark at room temperature. After 5 min, 0.6 mL of 10% (w/v) AlCl_3_ was added, and the mixture was incubated for more 6 min. Following incubation, 2 mL of 1 M NaOH and 2.1 mL of distilled water were introduced. The mixture was filtered through paper, and its absorbance was measured at 510 nm using a spectrophotometer (T70 + UV/VIS spectrophotometer, PG Instruments, UK). The result was expressed as “mg routine/g dry matter.”

### 2.13 Textural Analyses

The textural attributes, like skin strength (Newton) and elasticity (mm distance), of the dried grapes (both C102 and C103), were assessed using an analyzer (Stable Micro Systems, TA‐XT Plus, UK). Measurements were conducted in five replicates.

### 2.14 Scanning Electron Microscopy (SEM)

SEM images were taken to examine structural changes resulting from carbonic maceration pretreatment in three sample types: C101, C102, and C103.

### 2.15 Statistical Analysis

The Minitab Statistical Software (Minitab, State College, USA) was employed to design experiments and perform a statistical optimization analysis using RSM. To assess the significance of mean differences, a Tukey pairwise comparison test was conducted with a significance level of *p* ≤ 0.05. The results were presented as mean ± standard deviation.

## 3 Results and Discussion

This study aimed to examine the drying process of seedless grape samples and to evaluate how CM treatment and drying conditions affect the dried product's characteristics. To ensure adequate comparison, fresh Sultani grape samples were analyzed, and the color, a_w_, MC, titration acidity, pH, °Bx, TPC, TEAC, and TFC were determined, and the results were given in Table 4. The MC, aw, °Bx, TA, TPC, TEAC, and TFC of the fresh grapes are coincident with the previous literature findings (Candemir et al. 2023; Dincer 1996; Ismail 2005; Karacabey et al. 2020; Kedage et al. 2007).

**TABLE 4:** Physicochemical properties of fresh Sultani grapes.

Analyses	Fresh grape (C101)
a_w_	0.903 ± 0.003
MC	70.77 ± 0.056
TA	0.145 ± 0.003
pH	3.88 ± 0.004
°Bx	12.45 ± 0.212
L*	46.49 ± 3.353
a*	1.607 ± 1.046
b*	9.475 ± 1.579
C*	9.668 ± 1.501
hue	82.523 ± 9.934
TPC	8.462 ± 0.083
TEAC	12.045 ± 0.752
TFC	20.383 ± 2.691

*Note:* L*, Lightness; a*, rednesss; b*, yellowness; hue, hue value; C*, chroma.

Abbreviations: MC, moisture content (g H_2_O/100 g w.b.); TA, titratable acidity (%); TEAC, Trolox equivalent antioxidant capacity (mg TE/g DM); TFC, total flavonoid content (mg rutin/g DM); TPC, total phenolic compounds (mg GAE/g DM); a_w_, water activity.

### 3.1 The Changes in the Physicochemical and Functional Properties of Dried Grapes Depending on Process Conditions

Carbonic maceration treatment and the following drying were carried out for grape samples according to the previously determined design table (Table 2). Measurement of color parameters, drying time, rehydration ratio, textural qualities, TFC, TPC, TEAC, and HMF was performed. However, only the corresponding results for drying time, elasticity, TEAC, and hue angle value are given in Table 5, since the model performance parameters were only found to be acceptable for these variables (*R* ^2^ > 0.70 and *p* value of the lack‐of‐fit test > 0.05) to gain access to the optimization step (Table 6).

**TABLE 5:** Measured values of the examined responses.

Run number^a^	Drying time (min)	Elasticity (mm)	Total antioxidant capacity (mg TE/g DM)	Hue angle value
1	1050	7.53	3.26	56.28
2	520	6.66	6.59	50.44
3	435	7.75	7.53	62.57
4	960	7.96	3.25	48.77
5	269	7.56	7.70	66.48
6	421	7.46	8.07	64.33
7	504	8.16	3.86	59.31
8	469	6.39	2.75	60.90
9	248	6.49	7.70	62.60
10	240	5.54	7.70	65.37
11	515	4.25	9.92	58.81
12	600	6.19	6.49	60.27
13	495	4.88	5.45	63.12
14	1125	5.50	6.39	55.95
15	1066	3.99	3.43	51.46
16	515	4.58	2.77	61.47
17	255	7.59	5.34	59.39
18	500	4.22	3.98	60.26
19	525	5.78	3.07	56.10
20	571	4.98	2.73	56.67
21	519	6.35	5.46	58.95
22	875	5.30	3.12	60.33
23	765	5.40	5.22	60.69
24	242	6.51	8.34	68.51
25	420	7.32	6.30	66.11
26	555	6.62	5.00	55.59
27	270	6.87	5.56	64.49

^a^ Randomly distributed.

**TABLE 6:** Performance parameters and set of coefficients of the developed models for corresponding responses.

Variables^a^	Drying time (min)	Elasticity (mm)	Total antioxidant capacity (mg TE/g DM)	Hue angle value
β_0_	7706***	11.76***	64.5**	0.3***
β_1_ (X_1_) CMP	1980^ns^	—	‐60^ns^	−93.1^ns^
β_2_ (X_2_) CMT	—	−0.435^ns^	−0.2607**	−0.1897**
β_3_ (X_3_) CMt	—	—	−0.331^ns^	2.56^ns^
β_4_ (X_4_) DryT	−174.1***	−0.683*	−1.564**	1.014***
β_11_ (X_1_X_1_)	−5424*	—	211.3**	229*
β_22_ (X_2_X_2_)	—	0.001906*	0.00444*	—
β_33_ (X_3_X_3_)	—	—	0.02020*	—
β_44_ (X_4_X_4_)	0.986**	—	0.01222*	—
β_13_ (X_1_X_3_)	—	—	−1.530*	—
β_24_ (X_2_X_4_)	—	0.00495*	—	—
β_34_ (X_3_X_4_)	—	—	—	−0.0356*
Model	***	***	**	***
*R* ^2^	93.99	71.39	70.03	72.98

*Note:* a: Variables: *β*_0_ is the constant coefficient; *β*_*i*_ is the linear coefficient (main effect); *β*_*ii*_ is the quadratic coefficient; *β*_*ij*_ is the two factors' interaction coefficient. ns, not significant (*p* > 0.05); *, significant at *p* ≤ 0.05; **, significant at *p* ≤ 0.01; ***, significant at *p* ≤ 0.001—the values with a significance level less than 0.1 were removed from the model using backward elimination.

Abbreviations: CM temperature; CMt, CM duration; CMT, DryT, drying temperature (oven temperature) are represented by the subscripts 1, 2, 3, and 4, accordingly; CMP, CM pressure.

According to the experimental results, the responses, which included drying time, elasticity, total antioxidant capacity, and hue angle value, varied greatly based on the process parameters. To put it another way, those answers were considerably impacted by the process conditions under study, albeit to differing degrees. As far as we know, there has not been reported any data about the investigation of the effect of CM before drying on the drying performance and quality characteristics of seedless Sultani grapes. On the other hand, different pretreatment techniques focusing on grape drying have been reported in the literature, such as sodium hydroxide, ethyl oleate, potassium carbonate, abrasion, water blanching, ohmic heating, pulsed electric field, and ultrasound (Giuseppina Adiletta et al. 2015; Celik 2019; Dev and Raghavan 2012; Esmaiili et al. 2007; Khiari et al. 2019; Patidar et al. 2021; Salengke and Sastry 2005; Zemni et al. 2017). Those reports were useful to discuss the results of the current study. Furthermore, in the literature, there are CM pretreatment studies applied before drying for different foods. Vegetables and fruits, such as potatoes, plums, red grapes, strawberries, ginger, tomatoes, and capia peppers, were pretreated with CM before drying. Thus, they can help to assess the parameter effect on the product characteristics and process performance criteria. Like our findings, a decrease in drying time was declared in those reports. The reduced time requirement for drying by the CM treatment has been attributed to structural distortions due to some chemical reactions (An et al. 2020; An et al. 2018; Liu et al. 2014; Ozcelik et al. 2022; Turgut et al. 2018b; Wang et al. 2014; Zhao et al. 2016).

Other significant models developed for the studies responses were corresponding to elasticity values, TEAC, and hue values of dried grape samples. According to the experimental design, the elasticity values of the dried samples varied between 3.99 mm and 8.16 mm (Table 4). TEAC of dried grape samples was in the range of 2.75 to 9.92 mg TE/g DM. When hue values were considered, it was seen that their lowest value was 48.77, and the highest one was 68.52. Similar to our results, numerous studies documented in the literature that hue values in the range of 49.2–63.90 varied depending on the variety and the pretreatments applied for grapes (Lydakis et al. 2003; Teker et al. 2023).

### 3.2 Modeling of Dependent Variables

The primary purpose was to optimize the CM pretreatment and the hot air‐drying conditions using RSM. For the optimization, moisture content, a_w_, rehydration ratio, color parameters, pH, °Bx, titration acidity, drying time, texture analyses, TPC, TEAC, HMF, and TFC were selected as the dependent variables. However, the statistical analysis indicated that only the corresponding models for drying time, elasticity, TEAC, and hue angle were significant (*p* ≤ 0.001). Thus, they were used for the optimization of the examined drying technique. Table 6 displays the model performance parameters and the related regression coefficients for each model. Backward elimination was employed to omit the nonsignificant model terms for better performance, especially to avoid overfitting. The models in Table 6 explained more than 70% of the variance in the responses as a function of the investigated parameters. This model performance parameter exceeded 90% for drying time.

The drying process prioritizes minimizing energy consumption and enhancing product quality. This makes process time the crucial dependent variable for drying‐related studies (Motevali et al. 2011), as well as for the current one. As can be seen from the developed model of drying time in Table 6, statistical significance was observed only for the linear term for drying temperature (DryT) and the quadratic terms of CMP and DryT. All remaining terms (linear terms of CMT and CMt, quadratic terms of CMT and CMt, and all interaction terms between all pairs of the studied parameters) were found to be nonsignificant terms and eliminated by the backward elimination method (*p* > 0.05). The outcome of the lack‐of‐fit test revealed that there was no problem (*p* > 0.05). Depending on the process parameters, process time for drying of grape samples varied from 240 to 1125 min (Table 5). That time requirement was 510 min for the control group. Thus, it can be concluded that for some design runs, a shorter process was achieved compared with the control one. This reduction in process duration may be caused by CM pretreatment. Gunes et al. (2005) reported that the dehydration of plant materials was affected when drying material was subjected to CM pretreatment. An acceleration in drying may be attributed to the structural influence of CM treatment. As carbon dioxide dissolves in plant tissue during pretreatment, it transforms into carbonic acid, leading to cracks, particularly on the fruit's exterior surface and skin. Thus, the molecular transfer of water from the inner side of plant material to the outer surface becomes easier (Liu et al. 2014; Turgut et al. 2018a).

Elasticity was another important parameter considered in the drying of grape samples in a convection oven following CM pretreatment. The elasticity model was able to explain more than 71% of the variation in this response (CM pretreatment conditions and drying temperature) based on process conditions. There was also no doubt about the lack of fit of the model (*p* > 0.05). Only the first‐order term of DryT, the second‐order term of CM temperature (CMT), and the interaction term between CMT and DryT were found to be statistically significant (*p* ≤ 0.05). During the hot air drying of most biological materials, including plants without any pretreatment, the resistance against water transfer depends on the cellular matrix behavior and phases in the structure. The structure becomes firm due to the rapid evaporation of moisture from the layers close to the surface. On the other hand, dissolved chemical substances, especially those with high molecular weights, are transferred to the surface layer during drying; as a result, those substances contribute to the rigidity of the structure. However, CM pretreatment makes the high molecular weight components of the cell wall (cellulose and hemicellulose) convert into smaller molecules (beta galacturonic acid, glucose, and arabinose). As a consequence, the cell wall collapses (Krall and McFeeters 1998; Turgut et al. 2018a). The resistance of the fruit structure to the applied external force reduces because of the breakdown of high molecular weight polymers and cell wall degradation (Turgut et al. 2018a). Thus, structural resistance may decrease in CM‐pretreated samples, and dried fruits may have a more flexible structure and higher elasticity.

Grapes are accepted as antioxidant‐rich fruits that contain resveratrol, flavanone, flavanols (quercetin, kaempferol, and myricetin), phenolic acids (hydroxycinnamic and hydroxybenzoic acids), and anthocyanins, all of which aid in free radical damage neutralization. In this extent, the antioxidant potential of the dried grape sample was under investigation, and it was found that the studied response, TEAC of seedless Sultani grapes, was highly affected by CM parameters (Giampieri et al. 2012). The determination coefficient showed that the model adequately explained the variation in the total antioxidant capacity depending on the CM treatment and drying conditions (Table 6). In the developed model, first‐order terms of DryT and CMT were significant (*p* ≤ 0.01). All second‐order terms in the model were found to be statistically significant (*p* ≤ 0.05). Only one of the interaction terms between CMP and CM time (CMt) showed a significant effect on TEAC values (*p* ≤ 0.1) (Table 6). The rest of the terms in the polynomial model were omitted by using backward elimination. Notably, there was no significant lack of fit (*p* > 0.05).

Another dependent variable analyzed in this study was hue value, which allows the observer to identify the surface color. It describes main colors like red, green, blue, and yellow (RGB) and related colors' luminance or darkness (Cantrell et al. 2010). The significance of color value has been emphasized in numerous drying experiments, especially on fruits such as kiwi, banana, and strawberries, and it has been highlighted more in the literature than other color parameters (Conti et al. 2014; Crecente‐Campo et al. 2012; Diamante et al. 2010; Nunes and Delgado 2012; Ornelas‐Paz et al. 2013; Wojdyło et al. 2009). The hue value emerged as the most well‐explained color value in this investigation, with a 73% explanation and no significant lack‐of‐fit issue (*p* > 0.05). Among the model terms, the first‐order terms of CMT and DryT, as well as the second‐order term of CMP, were statistically significant (*p* ≤ 0.05). A substantial interaction effect on hue value was only observed between CMt and DryT (*p* ≤ 0.05). All The remaining first and second‐order members, as well as their interactions, were excluded from the model (*p* > 0.05).

**FIGURE 1:**
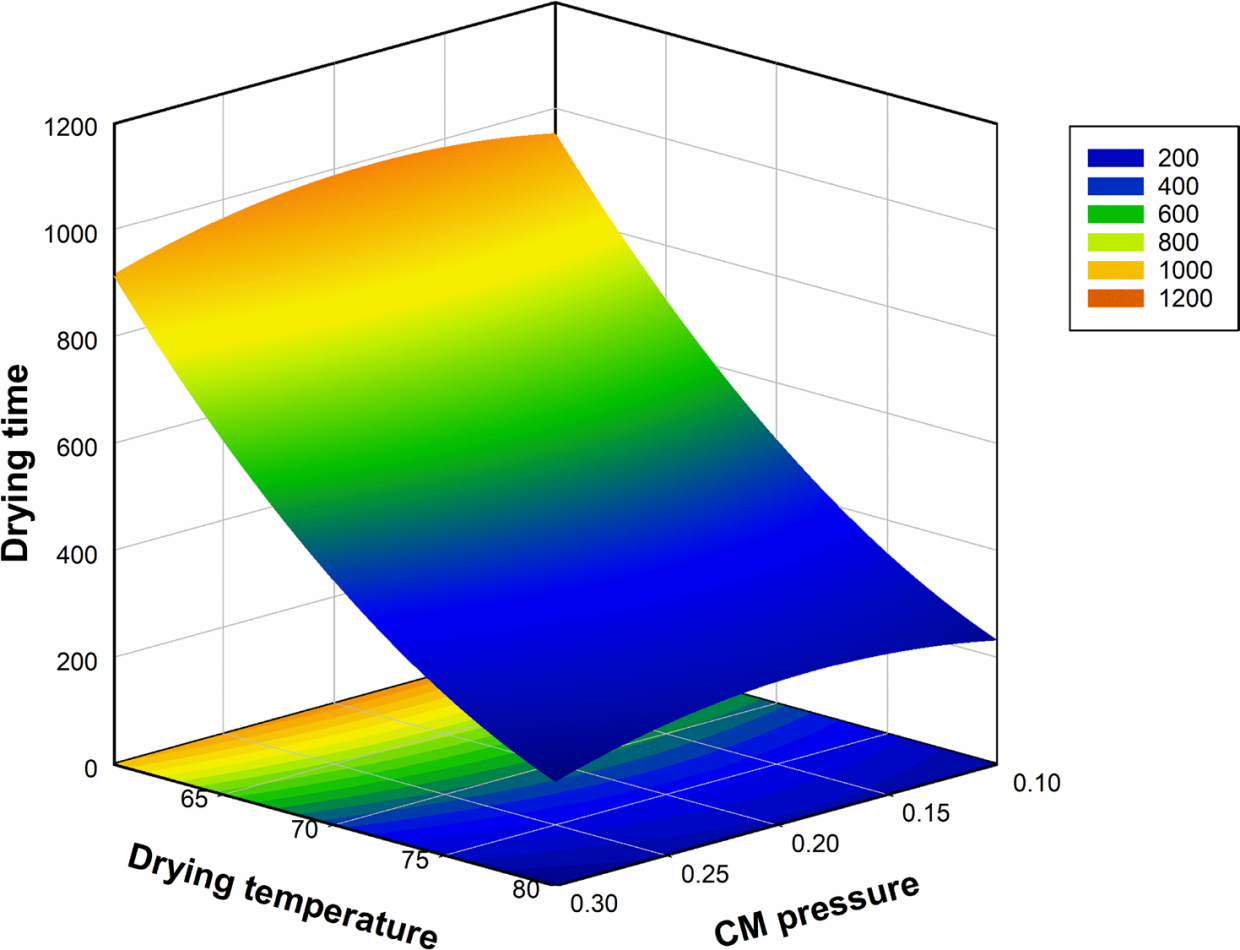
Influence of CMP (MPa) and DryT (°C) on the drying time (h) of grape samples.

To assess how CM pretreatment impacts the drying time of grape samples, Figure 1 was drawn as a function of CMP and DryT based on the corresponding model. There was almost a linear decrease in drying time with increasing drying oven temperature. The shortest drying time was obtained at the highest value of oven temperature in the studied range (80°C). In addition, it was determined that the CMP caused a variation in the drying time with a slight curvature trend, but the time requirement reduced to its lowest level at the highest pressure level of 0.3 MPa of CM pretreatment (Figure 1).

In the literature, the effect of DryT on the quality of Monukka seedless grapes was investigated. It was determined that the temperature had a significant effect on the process time (Xiao et al. 2010). Water vapor diffusion accelerates as the temperature of the drying chamber rises, and the high driving force of mass transfer increases the drying rate (Candemir et al. 2023). In the study carried out to determine the drying kinetics of grapes, it was determined that the diffusion coefficient increased with temperature (Azzouz et al. 2002). It is thought that the similar effect of temperature was also seen in the current study during the drying process of grapes, since the process time was drastically reduced when the temperature was elevated. Our results also indicated the presence of the CM effect on the drying process which caused shorter drying. This effect can be attributed to the structural changes in grapes during CM pretreatment. Similarly, Gunes et al. (2005) reported a shorter drying period because of some chemical reactions that appeared in plant tissue which occurred during carbonic maceration. Basically, medium pH decreases as a result of the presence of carbonic acid which is formed by carbon dioxide after its dissolution into the cell cytoplasm during CM treatment. These changes result in the decomposition of cell structure like cell walls collapsed, capillaries ruptured, and vacuoles ruptured. This type of structural modifications weakens the cell and/or membrane permeability to different extents and breaks down high molecular weight polymers into smaller ones resulting in an increase in water mobility and faster removal of water during the drying process (An et al. 2020; Liu et al. 2014). Similar reports about the permeability change of cell walls or membranes under the effect of CM treatment have been published by Krall and McFeeters (1998), Femenia et al. (1998), and An et al. (2020). Coinciding with our findings, those studies also stated that there was a considerable reduction in the mass transfer resistance, improvement in drying efficiency, and enhancement in the extraction performance of active compounds.

**FIGURE 2:**
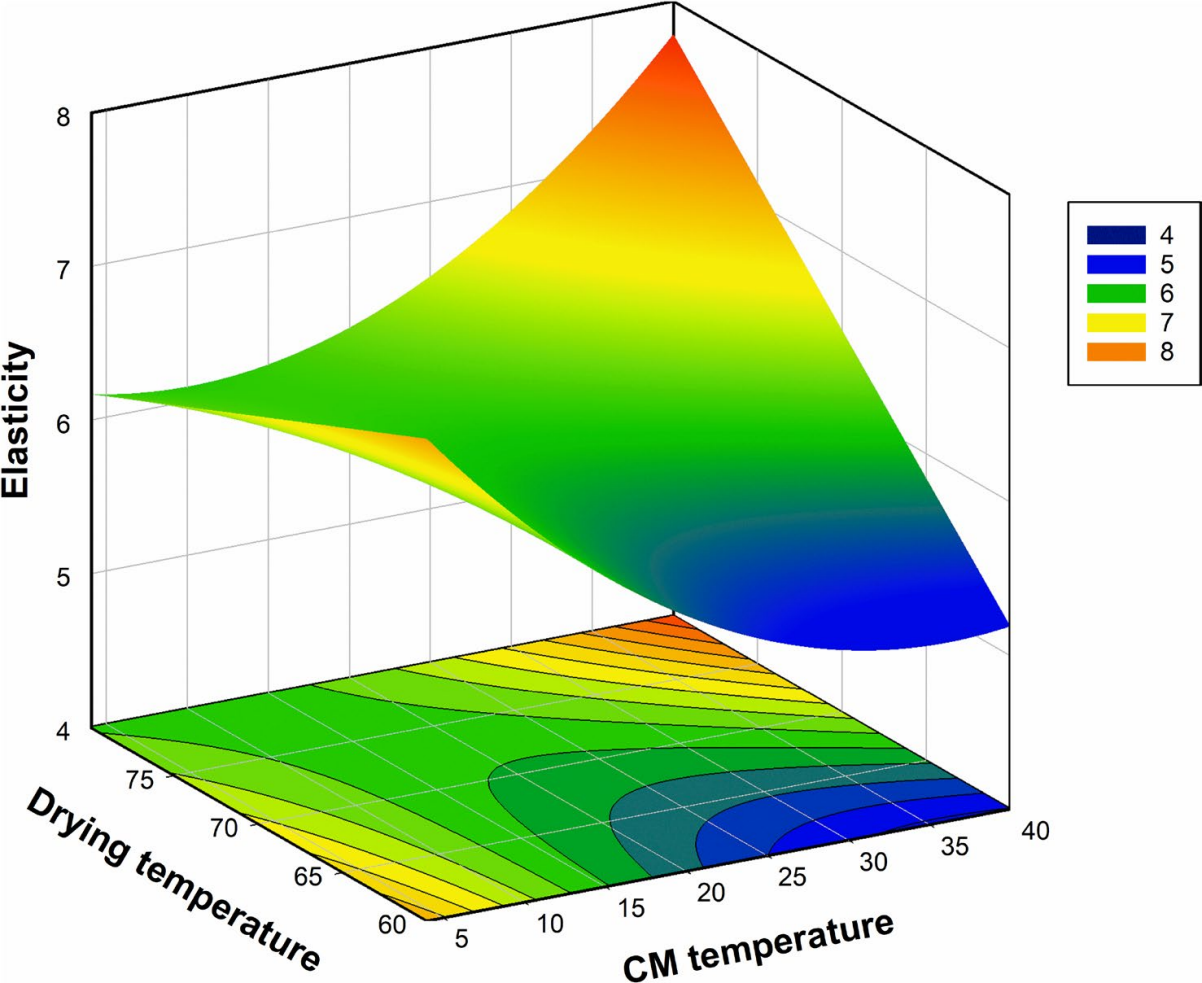
Influence of CMT (°C) and DryT (°C) on the elasticity (mm) of grape samples.

Elasticity is another investigated parameter of the dried grape samples. The influences of process conditions (DryT and CMT) on the elasticity value of dried grapes are shown in Figure 2. There was a limited effect of DryT on the elasticity values of samples when CM was applied at lower temperature levels of the studied range (Figure 2). However, as the pretreatment temperature increased up to its highest value, the influence of DryT became stronger, and the elasticity of dried samples increased (Figure 2). Another studied parameter, CMT was also affected the elasticity values. Similar to DryT, two different trends were observed for CMT (Figure 2). At lower levels of oven temperature, elasticity was adversely affected and decreased by CMT elevation. However, this trend shifted when the higher drying temperature was adjusted in a drying oven, and sample elasticity increased with a temperature rise of CM pretreatment. Figure 2 also indicates the presence of interaction between CMT and DryT. Increases in both process parameters intensified the increase in the elasticity value of the studied samples (Figure 2). CM‐dependent chemical reactions and the resultant structural changes may lead to a variation in the elasticity value of dried samples. Literature indicates the favorable effect of CM pretreatment on the water transfer rate, and this effect has been attributed to the physical and chemical changes (Liu et al. 2014; Wang et al. 2014) (Turgut et al. 2018a). It is well‐known that the water removal resistance of the drying tissue depends on the cellular matrix behavior and phases. Case hardening takes place especially on the outer surface because of the rapid moisture loss from outer layers in the case of hot air drying. Furthermore, dissolved high molecular weight substances in water are also transferred to the fruit surface layer. After water evaporation, those substances accumulate on the surface or in the layer close to surface, which contributes to the case hardening phenomena. For this reason, particularly the outer layers of the fruits gain a more solid structure and resist against external forces. Substances with a high molecular weight, such as cellulose and hemicellulose in the cell wall, are collapsed by the effect of CO_2_ and converted into smaller molecules (beta galacturonic acid, glucose, and arabinose). As a result, the cell wall collapses (Krall and McFeeters 1998; Turgut et al. 2018a). Due to the breakdown of high molecular weight polymers and damage to the cell wall, the resistance of the fruit structure to the applied force decreases. Thus, resistance to deformation may decrease in CM pretreated dried fruits, and they may have more flexible structures (Turgut et al. 2018a).

**FIGURE 3:**
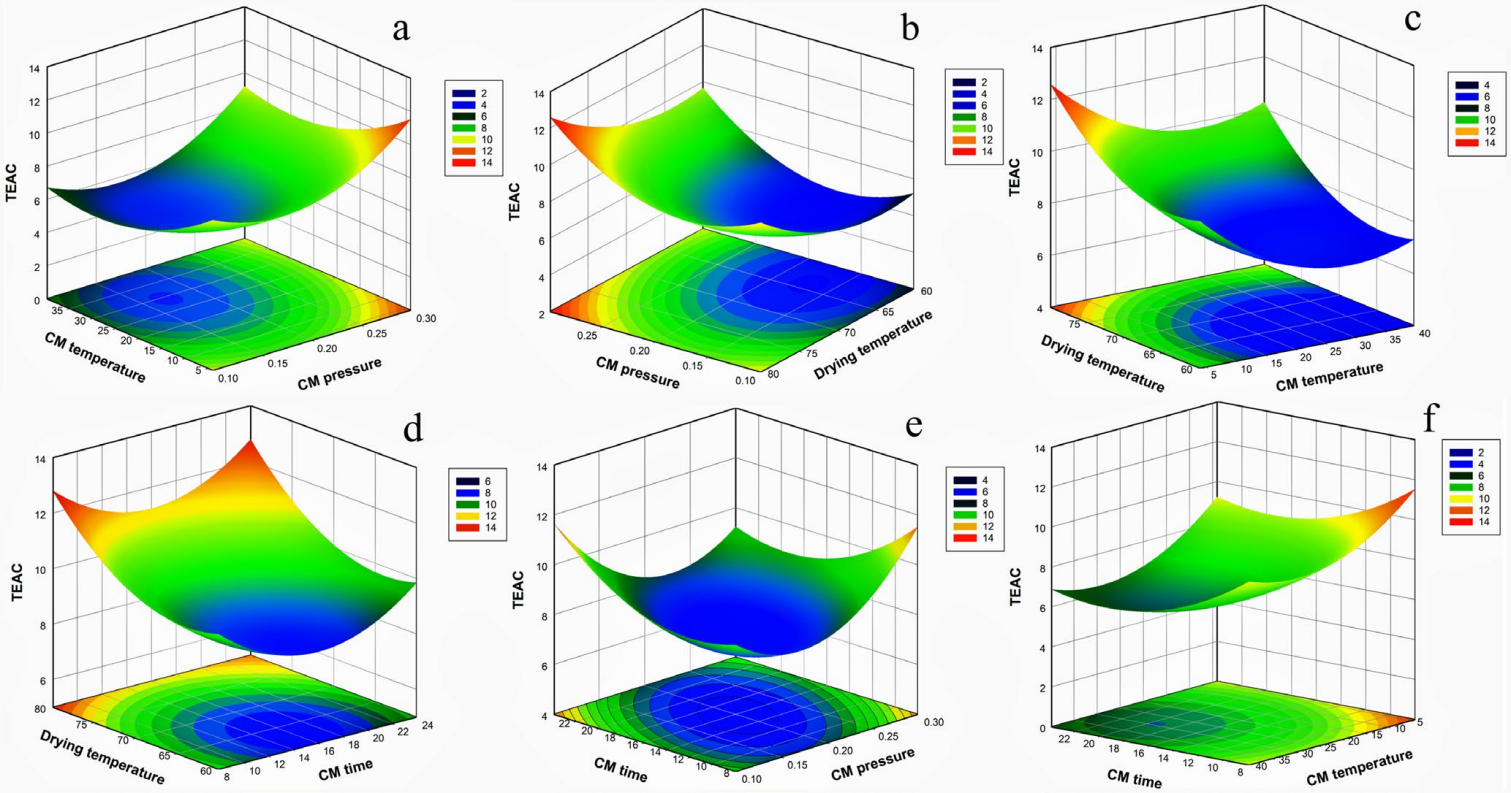
Influence of (a) CMT (°C) and CMP (MPa), (b) CMP (MPa) and DryT (°C), (c) DryT (°C) and CMT (°C), (d) DryT (°C) and CMt (h), (e) CMt (h) and CMP (MPa), (f) CMT (°C), and CMt (h) on TEAC (mg T.E/g DM) of dried grape with CM treatment.

According to model performance parameters and model terms, it is clear that the proposed pretreatment and the following drying temperature were all effective on the antioxidant potential of dried grape samples. Thus, a detailed investigation of the potential changes in TEAC value of samples depending on process conditions has been examined. Figure 3a–f displays the effects of CM process conditions (temperature, time, and pressure) and DryT on the TEAC values of grape samples. CMP and CMT affected the TEAC value as similar curved trends. In other words, any increase in those factors resulted in a decrease in TEAC value up to their corresponding moderate levels (around 20°C of CMT and 0.2 MPa of CMP). However, when those levels were exceeded, the TEAC value rose up again. Duan and Sun (2003) found that the solubility of CO_2_ gas in aqueous medium is directly proportional to pressure and inversely proportional to temperature. CO_2_ gas interacted more with the cells at low temperatures. In addition, CO_2_ gas lowers the cell pH, causing vacuoles to break down and the cell wall to be destroyed, resulting in the release of intracellular substances (Gunes et al. 2005). The main components of these substances are phenolics, flavonoids, and anthocyanins, which have antioxidant activities. Downey et al. (2007) found that phenolic and anthocyanin compounds were extracted better by applying acidification to grape extracts. There is a strong correlation between antioxidant activity and phenolic compounds (Deepa et al. 2006). Furthermore, CO_2_ application may inhibit key enzymes that may cause the oxidation of phenolic compounds (Gunes et al. 2005). As a result, the phenolic compounds were preserved, and subsequently, the antioxidant activity increased. Additionally, it is thought that low pH values of the CM treated samples enhanced the transition of phenolic compounds to the free form in the cell and are beneficial to this group of compounds against oxidation due in part to enzyme inhibition (Zhong et al. 2007). As a result, an increase in the TEAC value was observed. The highest TEAC results were obtained at the lowest level (around 4°C) of CMT and at the highest level (0.3 MPa) of CMP (Figure 3a). This may be associated with the dissolution of CO_2_ in the aqueous medium of plant cells, since it is well‐known that the dissolution of gaseous substances in liquid medium increases at lower temperatures and higher pressure levels. Variation in the TEAC value of dried grape samples depending on the CMP and DryT was displayed in Figure 3b. A curved shape change in TEAC values was observed in Figure 3b depending on CMP and DryT. However, it is worth mentioning that the influence of CMP on TEAC value was superior compared with the DryT effect. The highest antioxidant activity was observed at the highest level of DryT in the studied range. This favorable effect of oven temperature level may be attributed to the acceleration in the drying process. Because an increase in the DryT causes a decrease in the drying time, it dries faster, and the substances having antioxidant activity are less exposed to high temperatures and related oxidation reactions. As shown in Figure 3c, an increase in TEAC values was observed at low CMT levels and high DryTs. The effects of CMt and DryT on the TEAC value are shown in Figure 3d. Regardless of the drying temperature, the TEAC value decreased with an increase in the pretreatment time up to around 15 h, but a further increase in process time resulted in a trend change in TEAC value, and it started to rise to reach a certain level where the highest treatment time was attained. An increase in the DryT caused an increase in the TEAC values. Accordingly, drying at high temperature after short‐term CM maximized the TEAC values of the samples. In Figure 3e, it was determined that the TEAC value increased with increasing time at low CMP value and decreased for long processing times at high CMP values. Both CMt and CM temperatures had independent effects on the TEAC value. The TEAC value increased as CMT and time decreased (Figure 3f).

**FIGURE 4:**
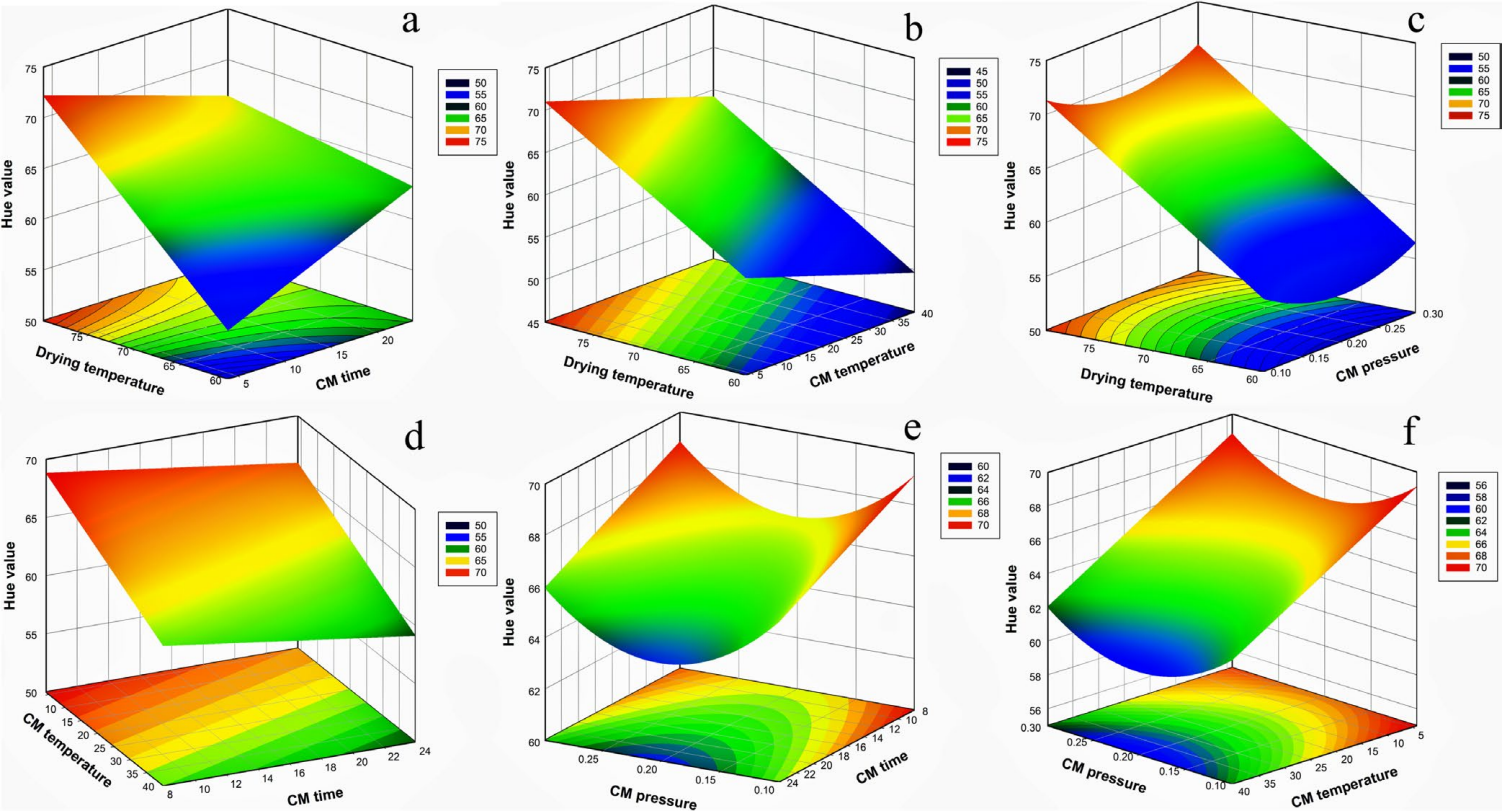
Influence of (a) DryT (°C) and CMt (h), (b) DryT (°C) and CMT (°C) and (c) DryT (°C) and CMP (MPa), (d) CMT (°C) and CMt (h), (e) CMP (MPa) and CMt (h), (f) CMP (MPa) and CMT (°C) on hue value of dried grape with CM treatment.

The studied factors (temperature, time, and pressure of CM treatment and oven drying temperature) were included in the developed model of hue value as different terms in that equation. As discussed above, according to the results given in Table 6, some variables are linear terms, some of them are quadratic, and one is an interaction term. Therefore, all possible pairs of independent variables and the corresponding hue value response surface graphs are drawn and given in Figure 4a–f. The corresponding hue value was in the range of 50–80 in those figures. In other words, generally, the skin color of dried grape samples was around the corresponding red tones and changed in this color range (hue value range of 0–120) but toward the fresh color of green, which started from a hue value of 120. Thus, an increase in hue value was evaluated as the skin color change of dried grape from dark tones (a characteristic result of the most of the drying processes) to lighter ones. Figure 4a demonstrates how the hue value changed depending on DryT and CMt. It is seen from Figure 4a that the change in hue value was linear with both parameters, and its highest value was achieved when CMt of pretreatment was at its shortest level and DryT was at the highest one. The Hue value variation of dried grapes with CMT and DryT is shown in Figure 4b. The characteristic linear trend of hue value change was identical for both independent variables but in different manners. In other words, hue value decreased as DryT decreased and CMT increased (Figure 4b). Curvature effects of the CM pressure on the hue value were seen from Figure 4c. An increase in the pretreatment pressure caused a decrease in hue value up to its pressure level range of 0.15 to 0.20 MPa, but once those moderate pressure levels were exceeded, hue value again started to increase, as seen in Figure 4c. All the Figures (Figure 4a,d,e), in which CMt was one of their independent variables, displayed the linear variation in hue value of dried samples with that variable, but in an adverse direction. In other words, this statement meant that hue value decreased when CMt increased, except for the linear trend of CMt observed when DryT was at its lowest level (Figure 4a). From the results, it was understood that the change of CM pressure and time on hue value was not as sharp as that observed under the effect of CMT and DryT. It was thought that this was mainly due to the link between treatment temperature‐dependent CO_2_ solubility change and the following oven temperature‐related drying rate change. Because CO_2_ solubility increases at low CM temperatures, it is more effective on product structure. In other words, as the temperature decreased, the CO_2_ gas penetrated the cell more effectively, reducing the intracellular and cytoplasm pH and causing structural degradation. Furthermore, CO_2_ treatment inhibited the activity of enzymes promoting oxidation, such as polyphenol oxidase and peroxidase (Gunes et al. 2005).

CM pretreatment has a positive effect on the color as it provides a short drying time and prevents browning reactions (Zhao et al. 2016). It has been reported that golden yellow is the preferred hue for dried grapes (FAO and OIV 2016). Browning reactions occur when the drying process takes a long period. As a result, color browning begins. To prevent this mechanism, the color is bleached by SO_2_ (sulfur dioxide) treatment. SO_2_ prevents browning by inhibiting the polyphenol oxidase enzyme in the matrix (Esmaiili et al. 2007). However, due to the fact that SO_2_ is a chemical substance, the bleaching effect in this study was obtained naturally through CM pretreatment. These investigations in the literature indicate that it corresponds to Figure 4. Color preservation is improved because CO_2_ activity is more active at low CM temperatures. As a result, the Hue value linearly rises with the DryT and approaches the yellow +b* (yellow) color value.

### 3.3 Model Optimization of Dependent Variables

To figure out the ideal conditions (CM temperature, time, pressure, and drying temperature) of the CM pretreatment and following drying of the grape samples, the models corresponding to the drying time, elasticity, TEAC, and Hue (h) value were used.

In order to determine the optimum conditions, the drying time was minimized, whereas the elasticity, hue, and TEAC values were maximized. According to the optimization analysis, DryT, CMt, CMP, and CMT were 77°C, 8 h, 0.3 MPa, and 4°C, respectively. The grape samples were processed under the optimum conditions, and drying was finished in 390 min. Without CM treatment, the control group was also dried at the optimal drying temperature of 77°C. Total drying time for the control group was measured as 510 min. As a result, the drying time was reduced by 24%. In other words, a significant enhancement in the drying process was achieved, and CM caused a faster drying process. Physical properties, bioactive contents, and related function potential were determined. The results of dried samples with (C102) and without the CM pretreatment (C103) are given in Table 7. According to these results, there was not any significant difference in the moisture content, water activity, and°Bx values of C102 and C103 (*p* > 0.05). As an expected result of CM treatment, pH should be lowered, and simultaneously, titratable acidity should be increased because the cell pH decreases with CM pretreatment (An et al. 2018). However, compared with the control group results (C102), the pH value of the CM‐treated samples was significantly higher, and titratable acidity was lower (*p* ≤ 0.05) (Table 7). As it is well known that CO_2_ application is inversely proportional to temperature and directly proportional to pressure (Duan and Sun 2003). Similarly, the highest pressure and lowest temperature levels were determined as the optimal conditions of CM treatment. Thus, it was expected that CO_2_ gas would be more effective on the samples and, as a result, the cell pH would remain at lower levels as titratable acidity was higher. However, in this study, CM pretreatment did not result in the expected results. This may be associated with the volatilization of carbonic acid during the drying step because carbonic acid (H_2_CO_3_), which is the dissolved form of CO_2_ gas in water, is volatile. Therefore, during the drying process, the dissolved and/or undissolved CO_2_ in the product structure moves away from the structure, and as a result, the CO_2_‐induced pH change did not develop in the expected direction (Turgut et al. 2018a). Similarly, the change in pH of grape juice with CO_2_ application was examined, but no significant change was observed in pH (Gunes et al. 2005) (Table 7).

**TABLE 7:** Analysis findings of dried grapes with and without CM pretreatment.

Analyses	Control (C103)	Optimum (C102)
a_w_	0.56 ± 0.060^a^	0.63 ± 0.059^a^
MC	15.88 ± 7.22^a^	16.84 ± 1.77^a^
°Bx	2.95 ± 0.070^a^	2.50 ± 0.141^a^
pH	4.09 ± 0.064^b^	4.37 ± 0.007^a^
TA	1.28 ± 0.038^a^	1.07 ± 0.006^b^
L*	38.91 ± 3.03^a^	46.62 ± 4.45^a^
a*	13.24 ± 0.86^a^	10.65 ± 1.07^a^
b*	21.11 ± 2.42^a^	22.31 ± 3.02^a^
C*	25.00 ± 2.51^a^	24.73 ± 3.19^a^
Hue	57.50 ± 1.65^b^	64.41 ± 0.83^a^
ΔE	10.82 ± 0.02^a^	14.01 ± 4.05^a^
TPC	2.79 ± 0.030^b^	3.35 ± 0.137^a^
TEAC	1414.36 ± 3.99^b^	1841.86 ± 1.97^a^
HMF	3.57 ± 0.00^b^	2.65 ± 0.00^a^
TFC	0.34 ± 0.074^a^	0.47 ± 0.045^a^
Elasticity	6.98 ± 3.02^a^	6.25 ± 2.24^a^
Skin strength	467.0 ± 158.4^a^	271.3 ± 99.6^a^

*Note:* L*, lightness; a*, redness; b*, yellowness; hue, hue value; C*, chroma; elasticity (mm), and skin strength (g force). Different letters in the same row are significantly different (*p* ≤ 0.05).

Abbreviations: HMF, hydroxymethylfurfural (mg HMF/100 DM); MC, moisture content (g H_2_O/100 g w.b.); TA, titratable acidity (%); TEAC, Trolox equivalent antioxidant capacity (μmol TE/g DM); TLC, total flavonoid content (mg routine/g DM); TPC, total phenolic compounds (mg GAE/g DM); a_w_, water activity.

While the differences in L*, a*, b*, C*, and ΔE values of the dried samples with and without CM pretreatment were not statistically significant (*p* > 0.05), there is only a statistically significant difference between the Hue values (*p* ≤ 0.05) (Table 7). The higher hue value of CM pretreated grapes can be considered a color improvement of the dried sample, since a higher hue value measured in the current study (50–70) means that the skin color gets close to that desired one. The desired color of grapes is golden yellow (FAO and OIV 2016). With the progress of ripening in the grain, chlorophyll pigment disappears in white and yellow grape varieties, and the skin color changes from green to white‐yellow. These yellow colors are composed of quercetin or quercitrin, a flavonyl pigment. When oxidized, flavones form amber yellow or amber red colors (Söylemezoğlu 2003). In addition, CO_2_ application inactivates enzymes, such as polyphenol oxidase and peroxidase, which can cause oxidation (Gunes et al. 2005). With the application of CM, the drying rate increased, and in this case, it played an active role in limiting browning reactions. Subsequently, it is seen that the product that interacts less with temperature has less discoloration, so it reaches the desired final product. As with color parameters, textural properties are decisive physical factors for product quality. The texture properties (elasticity and puncture test) of the optimum samples did not differ from those of the control samples (*p* > 0.05). Thus, looking at the color and texture results, it can be said that the CM pretreatment applied in this study did not have any adverse effects compared with the control samples (Table 7).

Prolonged exposure to high temperatures during the drying process causes a loss of nutritional and sensory quality. In the food industry, besides bringing the physical properties of the product to the desired criteria, it is also aimed at protecting its bioactive compounds. Considering the bioactive compounds of dried grape samples with and without CM pretreatment, TPC and TEAC values of C102 samples were significantly higher (*p* ≤ 0.05) than C103, unlike a difference in their TFCs. Those were identical (*p* > 0.05) (Table 7). During the grape drying process, phenolic compounds may be lost due to enzymatic activity and oxidation (Breksa et al. 2010); (Bennett et al. 2011) (Serio et al. 2014). In addition to this view, it is possible to extract these compounds more easily with pretreatments applied during the drying process (Carranza‐Concha et al. 2012). The CM pretreatment temperature was low (4°C) in grapes dried under optimum conditions. The gases are more active at low temperatures. Therefore, it is thought that phenolic compounds are better preserved, and the amount extracted increases in the optimum samples. During fruit drying, cell walls deform and collapse. Although the release of oxidative and hydrolytic enzymes in fruits is triggered, these enzymes are inactivated at high drying temperatures, and phenolic substances are preserved (Chang et al. 2006). The high total phenolic content in the CM pretreated samples led to their high antioxidant substance content (TEAC). The antioxidant effect is not only dependent on phenolics but also varies according to the duration of the applied process and the method used (Serio et al. 2014). During heat treatments, such as hot air drying, high molecular weight antioxidant compounds undergo thermal decomposition and turn into more active small antioxidants. In this way, bound phenolic substances are released and antioxidant activity increases (Maillard and Berset 1995). On the other hand, compounds such as HMF that cause undesirable effects on human health are formed in products that are dried at high temperatures. Thus, the determination of HMF content in dried products is an important quality index (Jorge et al. 2018). The amount of HMF in CM pretreated dried grape was significantly lower than that in control samples. As the drying time is shortened by CM pretreatment, nonenzymatic browning reactions are reduced. Consequently, HMF formation is decreased (Çağlarırmak 2006).

Considering all the results obtained, CM pretreatment was able to produce dried grapes at least as well as the control samples, and even with superior properties, in a much shorter time. CM processing before drying is promising as an important alternative pretreatment both in the drying industry and in scientific studies, thanks to the fact that it does not require a chemical application and the system is environmentally friendly.

### 3.4 Images Captured Using Scanning Electron Microscope

**FIGURE 5:**
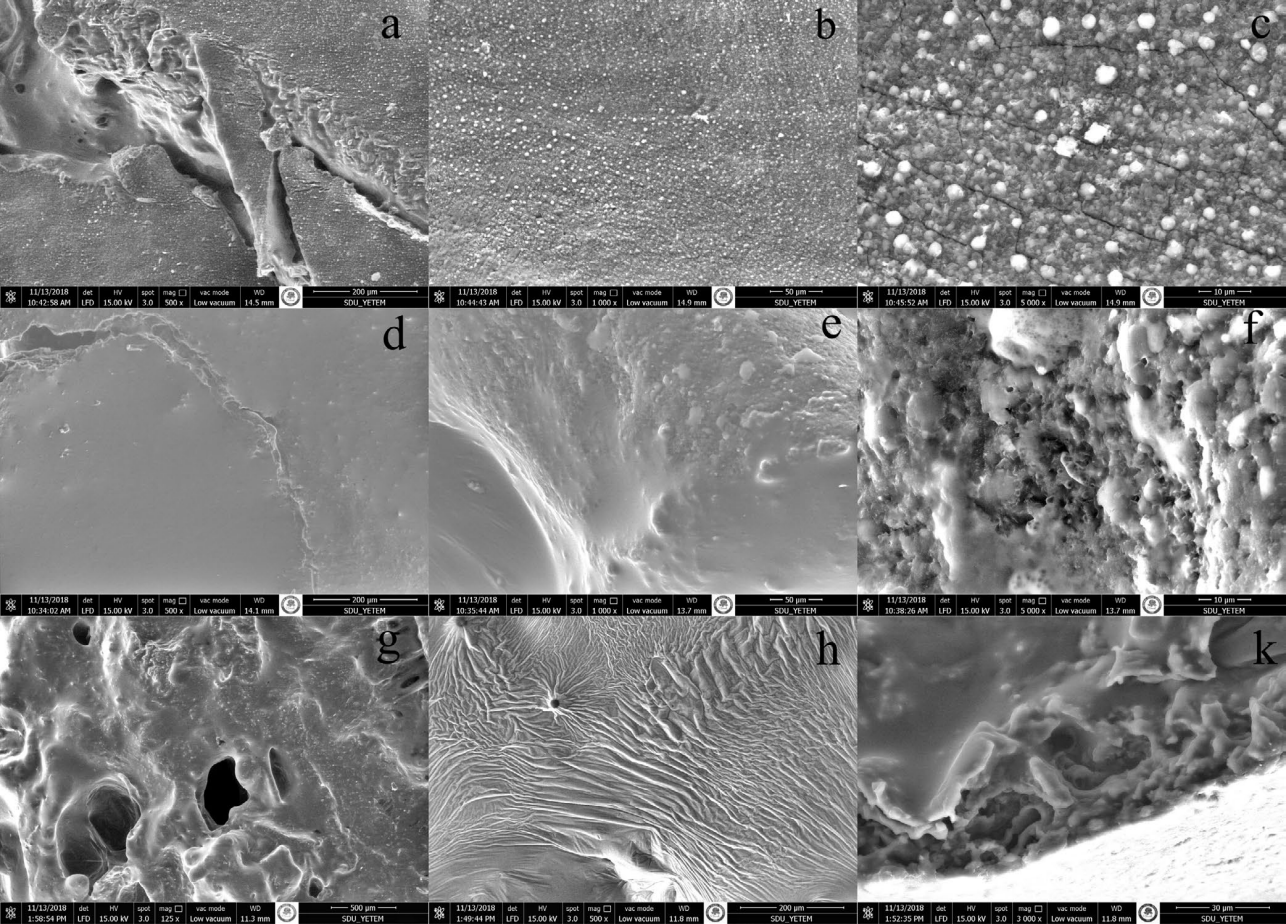
SEM image of dried samples (a): C102 exterior section (200 μm), (b): C102 exterior section (50 μm), (c): C102 exterior section (10 μm), (d): C103 exterior section (200 μm), (e): C103 exterior section (50 μm), (f): C103 exterior section (10 μm), (g): C102 cross section (500 μm), (h): C103 cross section (200 μm), (k): C103l cross section (30 μm).

To investigate the impact of CM pretreatment effect on the microstructure, image analysis was performed with a scanning electron microscope. Surface imaging was performed on grape samples dried under optimum conditions (C102) and control (C103) (Figure 5). Structural changes have been figured out in microscopic dimensions caused by the treatment processes. The surface images of the optimum and control samples were examined. It was determined that while the carbonic maceration pretreatment produced sugar crystals as a result of cracks and leakage on the surface in the optimum sample, it preserved the structure of the surface in the control sample. While it was determined that the pore structure was seen by opening micro channels with the effect of CO_2_ gas in the optimum sample, it was determined that it remained in a rigid structure in the control sample (Figure 5). The waxy layer is important because it affects moisture permeability in some fruits. The sugar in the structure of the treatment of the samples CM pretreatment occurs on the surface in the form of crystal particles.

In a study on the drying of seedless grapes, it was observed that the cell walls of the samples (control) that were not pretreated with CM remained tight, rigid, and less porous. In addition, it was observed that holes were formed in the CM pretreated samples. On the other hand, the applied CMP opened microchannels and facilitated the evaporation of water (Ozcelik et al. 2022). Following CM treatment, An et al. (2020) stated that the increased and expanded pore structures in Chinese Ginger are beneficial for enhancing the internal diffusion and evaporation of moisture. According to An et al. (2018), it was determined that the CM treatment caused changes in the structure of the plum. Besides, CO_2_ gas caused cell wall destruction and disruption of the middle lamellae. After the CM pretreatment, the cell wall folds became prominent, and the porous structure increased. In addition, it was determined that the pores enlarged and expanded with the increase in CO_2_ gas pressure.

Aguilera et al. (1987) implemented various pretreatments to the drying of Sultani grapes and evaluated their microstructure with SEM compared with the control. Accordingly, it was observed that the wax structure in the skin of the grape was removed with the pretreatments, causing multiple microcracks on the surface. In this way, they stated that the moisture transfer rate increased, and the drying rate increased.

## 4 Conclusions

In this study, the potential of applying CM as a pretreatment in the drying of grapes using the conventional drying method was investigated. The aim was to shorten the drying time of the CM process, thus preventing the deterioration caused by temperature and oxidation applied during the drying process to a certain extent. In this context, the response surface method created an experimental design that included three factors (temperature, time, and CO_2_ pressure). The optimum DryT, CMt, CMP, and CMT were 77°C, 8 h, 0.3 MPa, and 4°C, respectively. Considering drying times, 24% of the processing time was saved at the optimum conditions (with CM pretreatment) compared with the time requirement for the control (without CM pretreatment). No adverse effects were detected on the quality characteristics such as TPC, TFC, TEAC, color, and texture of the dried grape samples treated by CM application. TPC, Hue, and TEAC values of the samples dried under optimum conditions were higher than those of the control group (*p* ≤ 0.05), and the L*, a*, b*, ΔE, and texture were identical to the control (*p* > 0.05). In addition, the HMF value of the control samples (without CM pretreatment) was higher than that of the optimum samples (CM applied) (*p* ≤ 0.05). Consequently, the analysis results of the dried samples obtained are convincing and promising in terms of using CM as a pretreatment before drying. In the light of the findings in the current study and the reports given in the literature, it is worth saying that the carbonic maceration pretreatment before the drying process to enhance the drying performance and quality parameters of products provides a potential to adopt it as an industrial application. But it should be added that the published data is not enough to evaluate it totally in industrial drying applications. Thus, more effort is required to investigate its different aspects and applications for different plant materials and with different drying techniques.

## Author Contributions

**Nursaç Akyol Kuyucuoğlu:** formal analysis (equal), investigation (equal), methodology (equal), writing – original draft (equal). **Muhammed Mustafa Özçelik:** formal analysis (equal), investigation (equal), methodology (equal), writing – original draft (equal), writing – review and editing (equal). **Merve Seçil Bardakçı:** formal analysis (equal), investigation (equal), methodology (equal), writing – original draft (equal), writing – review and editing (equal). **Erdoğan Küçüköner:** supervision (equal). **Erkan Karacabey:** funding acquisition (equal), project administration (equal), supervision (equal), writing – review and editing (equal).

## Conflicts of Interest

The authors declare no conflicts of interest.

## Data Availability

The data supporting the results of this study can be obtained by contacting the corresponding author.

## References

[fsn370066-bib-0001] Adiletta, G. , P. Russo , W. Senadeera , and M. Di Matteo . 2016. “Drying Characteristics and Quality of Grape Under Physical Pretreatment.” Journal of Food Engineering 172: 9–18. 10.1016/j.jfoodeng.2015.06.031.

[fsn370066-bib-0002] Adiletta, G. , W. Senadeera , L. Liguori , A. Crescitelli , D. Albanese , and P. Russo . 2015. “The Influence of Abrasive Pretreatment on Hot Air Drying of Grape.” Food and Nutrition Sciences 6: 355–364.

[fsn370066-bib-0003] Aguilera, J. , K. Oppermann , and F. Sanchez . 1987. “Kinetics of Browning of Sultana Grapes.” Journal of Food Science 52, no. 4: 990–993.

[fsn370066-bib-0004] Altindisli, F. , and F. Ozsemerci . 2013. “Efficacy Evaluation of RAK 2 PRO Dispensers Against Lobesia Botrana on Sultani Cekirdeksiz Grapes in Turkey.” IOBC wprs Bulletin 91: 219–225.

[fsn370066-bib-0005] An, K. , L. Wei , M. Fu , L. Cheng , J. Peng , and J. Wu . 2020. “Effect of Carbonic Maceration (CM) on the Vacuum Microwave Drying of Chinese Ginger (*Zingiber officinale* Roscoe) Slices: Drying Characteristic, Moisture Migration, Antioxidant Activity, and Microstructure.” Food and Bioprocess Technology 13, no. 9: 1661–1674.

[fsn370066-bib-0006] An, K. , J. Wu , D. Tang , et al. 2018. “Effect of Carbonic Maceration (CM) on Mass Transfer Characteristics and Quality Attributes of Sanhua Plum (*Prunus salicina* Lindl.).” LWT—Food Science and Technology 87: 537–545. 10.1016/j.lwt.2017.09.032.

[fsn370066-bib-0007] Araujo, P. W. , and R. G. Brereton . 1996. “Experimental Design III. Quantification.” TrAC Trends in Analytical Chemistry 15, no. 3: 156–163.

[fsn370066-bib-1006] Aydin, E. , S. S. Turgut , S. Aydin , et al. 2023. “A New Approach for the Development and Optimization of Gluten‐Free Noodles Using Flours From Byproducts of Cold‐Pressed Okra and Pumpkin Seeds.” Foods 12, no. 10: 2018. 10.3390/foods12102018. 37238836 PMC10216911

[fsn370066-bib-0008] Azzouz, S. , A. Guizani , W. Jomaa , and A. Belghith . 2002. “Moisture Diffusivity and Drying Kinetic Equation of Convective Drying of Grapes.” Journal of Food Engineering 55, no. 4: 323–330. 10.1016/S0260-8774(02)00109-7.

[fsn370066-bib-0009] Bai, J. W. , D. W. Sun , H. W. Xiao , A. S. Mujumdar , and Z. J. Gao . 2013. “Novel High‐Humidity Hot Air Impingement Blanching (HHAIB) Pretreatment Enhances Drying Kinetics and Color Attributes of Seedless Grapes.” Innovative Food Science & Emerging Technologies 20: 230–237. 10.1016/j.ifset.2013.08.011.

[fsn370066-bib-0010] Bennett, L. E. , H. Jegasothy , I. Konczak , D. Frank , S. Sudharmarajan , and P. R. Clingeleffer . 2011. “Total Polyphenolics and Anti‐Oxidant Properties of Selected Dried Fruits and Relationships to Drying Conditions.” Journal of Functional Foods 3, no. 2: 115–124. 10.1016/j.jff.2011.03.005.

[fsn370066-bib-0011] Breksa, A. P. , G. R. Takeoka , M. B. Hidalgo , A. Vilches , J. Vasse , and D. W. Ramming . 2010. “Antioxidant Activity and Phenolic Content of 16 Raisin Grape ( *Vitis vinifera* L.) Cultivars and Selections.” Food Chemistry 121, no. 3: 740–745. 10.1016/j.foodchem.2010.01.029.

[fsn370066-bib-0012] Çağlarırmak, N. 2006. “Ochratoxin A, Hydroxymethylfurfural and Vitamin C Levels of Sun‐Dried Grapes and Sultanas.” Journal of Food Processing and Preservation 30, no. 5: 549–562. 10.1111/j.1745-4549.2006.00088.x.

[fsn370066-bib-0013] Candemir, A. , G. Çalışkan Koç , S. N. Dirim , and R. Pandiselvam . 2023. “Effect of Ultrasound Pretreatment and Drying Air Temperature on the Drying Characteristics, Physicochemical Properties, and Rehydration Capacity of Raisins.” Biomass Conversion and Biorefinery 14, no. 16: 19623–19635. 10.1007/s13399-023-04269-8.

[fsn370066-bib-0014] Cantrell, K. , M. Erenas , I. de Orbe‐Payá , and L. F. Capitán‐Vallvey . 2010. “Use of the Hue Parameter of the Hue, Saturation, Value Color Space as a Quantitative Analytical Parameter for Bitonal Optical Sensors.” Analytical Chemistry 82, no. 2: 531–542. 20000770 10.1021/ac901753c

[fsn370066-bib-0015] Carranza‐Concha, J. , M. Benlloch , M. M. Camacho , and N. Martinez‐Navarrete . 2012. “Effects of Drying and Pretreatment on the Nutritional and Functional Quality of Raisins.” Food and Bioproducts Processing 90, no. C2: 243–248. 10.1016/j.fbp.2011.04.002.

[fsn370066-bib-1007] Chelladurai, S. J. S. , K. Murugan , A. P. Ray , M. Upadhyaya , V. Narasimharaj , and S. Gnanasekaran . 2021. “Optimization of Process Parameters Using Response Surface Methodology: A Review.” Materials Today: Proceedings 37: 1301–1304.

[fsn370066-bib-0016] Celik, M. 2019. “The Effects of Some Local Cultivars and Pretreatment Solutions on Drying Period and Raisin Grape Quality.” Erwerbs‐Obstbau 61, no. Suppl 1: 67–74.

[fsn370066-bib-0017] Chang, C.‐H. , H.‐Y. Lin , C.‐Y. Chang , and Y.‐C. Liu . 2006. “Comparisons on the Antioxidant Properties of Fresh, Freeze‐Dried and Hot‐Air‐Dried Tomatoes.” Journal of Food Engineering 77, no. 3: 478–485. 10.1016/j.jfoodeng.2005.06.061.

[fsn370066-bib-0018] Conti, S. , G. Villari , S. Faugno , G. Melchionna , S. Somma , and G. Caruso . 2014. “Effects of Organic vs. Conventional Farming System on Yield and Quality of Strawberry Grown as an Annual or Biennial Crop in Southern Italy.” Scientia Horticulturae 180: 63–71. 10.1016/j.scienta.2014.10.015.

[fsn370066-bib-0019] Crecente‐Campo, J. , M. Nunes‐Damaceno , M. A. Romero‐Rodríguez , and M. L. Vázquez‐Odériz . 2012. “Color, Anthocyanin Pigment, Ascorbic Acid and Total Phenolic Compound Determination in Organic Versus Conventional Strawberries (*Fragaria* × *Ananassa* Duch, cv Selva).” Journal of Food Composition and Analysis 28, no. 1: 23–30. 10.1016/j.jfca.2012.07.004.

[fsn370066-bib-1004] Czaplicka, M. , K. Parypa , A. Szewczuk , et al. 2022. “Assessment of Selected Parameters for Determining the Internal Quality of White Grape Cultivars Grown in Cold Climates.” Applied Sciences 12, no. 11: 5534. 10.3390/app12115534.

[fsn370066-bib-0020] Deepa, N. , C. Kaur , B. Singh , and H. C. Kapoor . 2006. “Antioxidant Activity in Some Red Sweet Pepper Cultivars.” Journal of Food Composition and Analysis 19, no. 6: 572–578. 10.1016/j.jfca.2005.03.005.

[fsn370066-bib-0021] Dev, S. R. , and V. G. Raghavan . 2012. “Advancements in Drying Techniques for Food, Fiber, and Fuel.” Drying Technology 30, no. 11–12: 1147–1159.

[fsn370066-bib-0022] Diamante, L. , M. Durand , G. P. Savage , and L. P. Vanhanen . 2010. “Effect of Temperature on the Drying Characteristics, Colour and Ascorbic Acid Content of Green and Gold Kiwifruits.” International Food Research Journal 17, no. 2: 441–451.

[fsn370066-bib-0023] Dincer, l. 1996. “Sun Drying of Sultana Grapes.” Drying Technology 14, no. 7–8: 1827–1838.

[fsn370066-bib-0024] Downey, M. O. , M. Mazza , and M. P. Krstic . 2007. “Development of a Stable Extract for Anthocyanins and Flavonols From Grape Skin.” American Journal of Enology and Viticulture 58, no. 3: 358–364.

[fsn370066-bib-0025] Duan, Z. , and R. Sun . 2003. “An Improved Model Calculating CO_2_ Solubility in Pure Water and Aqueous NaCl Solutions From 273 to 533 K and From 0 to 2000 Bar.” Chemical Geology 193, no. 3: 257–271. 10.1016/S0009-2541(02)00263-2.

[fsn370066-bib-0026] Esmaiili, M. , R. Sotudeh‐Gharebagh , K. Cronin , M. A. E. Mousavi , and G. Rezazadeh . 2007. “Grape Drying: A Review.” Food Reviews International 23, no. 3: 257–280.

[fsn370066-bib-0027] FAO and OIV . 2016. Table and Dried Grapes, 1–62. Food and Agriculture Organization of the United Nations and the International Organisation of Vine and Wine.

[fsn370066-bib-0028] Femenia, A. , E. S. Sánchez , S. Simal , and C. Rosselló . 1998. “Effects of Drying Pretreatments on the Cell Wall Composition of Grape Tissues.” Journal of Agricultural and Food Chemistry 46, no. 1: 271–276. 10.1021/jf9705025. 10554231

[fsn370066-bib-0029] Flanzy, C. , M. Flanzy , and P. Benard . 1987. La Vinification par Macération Carbonique. Editions Quae.

[fsn370066-bib-0030] Giampieri, F. , S. Tulipani , J. M. Alvarez‐Suarez , J. L. Quiles , B. Mezzetti , and M. Battino . 2012. “The Strawberry: Composition, Nutritional Quality, and Impact on Human Health.” Nutrition 28, no. 1: 9–19. 22153122 10.1016/j.nut.2011.08.009

[fsn370066-bib-0031] Gunes, G. , L. K. Blum , and J. H. Hotchkiss . 2005. “Inactivation of Yeasts in Grape Juice Using a Continuous Dense Phase Carbon Dioxide Processing System.” Journal of the Science of Food and Agriculture 85, no. 14: 2362–2368.

[fsn370066-bib-0032] Hidalgo, A. , and C. Pompei . 2000. “Hydroxymethylfurfural and Furosine Reaction Kinetics in Tomato Products.” Journal of Agricultural and Food Chemistry 48, no. 1: 78–82. 10.1021/jf990120u. 10637055

[fsn370066-bib-0033] Ismail, O. 2005. “Investigation the Effect of Potasium Carbonate Solutions on Drying of Sultana Grapes.” Journal of Engineering and Natural Sciences 1: 108–113.

[fsn370066-bib-0034] Jorge, A. , E. Sauer Leal , R. Sequinel , T. Sequinel , E. T. Kubaski , and S. Tebcherani . 2018. “Changes in the Composition of Tomato Powder (*Lycopersicon esculentum* Mill) Resulting From Different Drying Methods.” Journal of Food Processing and Preservation 42, no. 5: e13595.

[fsn370066-bib-0035] Karacabey, E. , T. Aktaş , L. Taşeri , and G. U. Seçkin . 2020. “Sultani Çekirdeksiz Üzüm Çeşidinde Farklı Kurutma Yöntemlerinin Kurutma Kinetiği, Enerji Tüketimi ve Ürün Kalitesi Açisindan Incelenmesi.” Tekirdağ Ziraat Fakültesi Dergisi 17, no. 1: 53–65.

[fsn370066-bib-0036] Kedage, V. V. , J. C. Tilak , G. B. Dixit , T. P. A. Devasagayam , and M. Mhatre . 2007. “A Study of Antioxidant Properties of Some Varieties of Grapes (*Vitis vinifera* L.).” Critical Reviews in Food Science and Nutrition 47, no. 2: 175–185. 10.1080/10408390600634598. 17364701

[fsn370066-bib-0037] Khiari, R. , H. Zemni , and D. Mihoubi . 2019. “Raisin Processing: Physicochemical, Nutritional and Microbiological Quality Characteristics as Affected by Drying Process.” Food Reviews International 35, no. 3: 246–298. 10.1080/87559129.2018.1517264.

[fsn370066-bib-1005] Kiliçkan, A. , N. Üçer , and I. Yalçin . 2010. “Moisture‐Dependent Physical Properties of Black Grape (*Vitis vinifera* L.) Seed.” Scientific Research and Essays 5, no. 16: 2226–2233.

[fsn370066-bib-0038] Krall, S. M. , and R. F. McFeeters . 1998. “Pectin Hydrolysis: Effect of Temperature, Degree of Methylation, pH, and Calcium on Hydrolysis Rates.” Journal of Agricultural and Food Chemistry 46, no. 4: 1311–1315. 10.1021/jf970473y.

[fsn370066-bib-0039] Krokida, M. K. , V. T. Karathanos , Z. B. Maroulis , and D. Marinos‐Kouris . 2003. “Drying Kinetics of Some Vegetables.” Journal of Food Engineering 59, no. 4: 391–403. 10.1016/S0260-8774(02)00498-3.

[fsn370066-bib-0040] Kupe, M. , B. Sayıncı , B. Demir , S. Ercisli , M. Baron , and J. Sochor . 2021. “Morphological Characteristics of Grapevine Cultivars and Closed Contour Analysis With Elliptic Fourier Descriptors.” Plants 10, no. 7: 1350. https://www.mdpi.com/2223‐7747/10/7/1350. 34371553 10.3390/plants10071350 PMC8309250

[fsn370066-bib-1002] LaPanse, A. J. , A. Krishnan , and M. C. Posewitz . 2021. “Adaptive Laboratory Evolution for Algal Strain Improvement: Methodologies and Applications.” Algal Research 53: 102122.

[fsn370066-bib-0041] Liu, L. , Y. Wang , D. Zhao , K. An , S. Ding , and Z. Wang . 2014. “Effect of Carbonic Maceration Pre‐Treatment on Drying Kinetics of Chilli (*Capsicum annuum* L.) Flesh and Quality of Dried Product.” Food and Bioprocess Technology 7, no. 9: 2516–2527. 10.1007/s11947-014-1253-6.

[fsn370066-bib-0042] Lundstedt, T. , E. Seifert , L. Abramo , et al. 1998. “Experimental Design and Optimization.” Chemometrics and Intelligent Laboratory Systems 42, no. 1–2: 3–40.

[fsn370066-bib-0043] Lydakis, D. , I. Fysarakis , M. Papadimitriou , and G. Kolioradakis . 2003. “Optimization Study of Sulfur Dioxide Application in Processing of Sultana Raisins.” International Journal of Food Properties 6, no. 3: 393–403.

[fsn370066-bib-0044] Maillard, M.‐N. , and C. Berset . 1995. “Evolution of Antioxidant Activity During Kilning Role of Insoluble Bound Phenolic Acids of Barley and Malt.” Journal of Agricultural and Food Chemistry 43, no. 7: 1789–1793.

[fsn370066-bib-1003] Martin‐Garcia, B. , S. Pimentel‐Moral , A. M. Gómez‐Caravaca , D. Arraez‐Roman , and A. Segura‐Carretero . 2020. “Box‐Behnken Experimental Design for a Green Extraction Method of Phenolic Compounds From Olive Leaves.” Industrial Crops and Products 154: 112741.

[fsn370066-bib-0045] Miraei Ashtiani, S.‐H. , M. Rafiee , M. Mohebi Morad , et al. 2020. “Impact of Gliding Arc Plasma Pretreatment on Drying Efficiency and Physicochemical Properties of Grape.” Innovative Food Science & Emerging Technologies 63: 102381. 10.1016/j.ifset.2020.102381.

[fsn370066-bib-0046] Motevali, A. , S. Minaei , and M. H. Khoshtagaza . 2011. “Evaluation of Energy Consumption in Different Drying Methods.” Energy Conversion and Management 52, no. 2: 1192–1199. 10.1016/j.enconman.2010.09.014.

[fsn370066-bib-0047] Nunes, M. , and A. Delgado . 2012. “Quality of Organic Compared to Conventionally Grown Strawberries at the Retail Level.” Paper presented at the VII International Strawberry Symposium 1049.

[fsn370066-bib-0048] Ornelas‐Paz, J. d. J. , E. M. Yahia , N. Ramírez‐Bustamante , et al. 2013. “Physical Attributes and Chemical Composition of Organic Strawberry Fruit (*Fragaria* × *ananassa* Duch, Cv. Albion) at Six Stages of Ripening.” Food Chemistry 138, no. 1: 372–381. 10.1016/j.foodchem.2012.11.006. 23265501

[fsn370066-bib-0049] Ozcelik, M. M. , G. Ozkan , and E. Karacabey . 2022. “Evaluation of Carbonic Maceration Effect as a Pre‐Treatment on the Drying Process of Strawberry.” Agriculture 12, no. 12: 2113. https://www.mdpi.com/2077‐0472/12/12/2113.

[fsn370066-bib-1001] Ozcelik, M. M. , S. Aydin , E. Aydin , and G. Ozkan . 2023. “Preserving Nutrient Content in Red Cabbage Juice Powder via Foam‐Mat Hybrid Microwave Drying: Application in Fortified Functional Pancakes.” Food Science & Nutrition 12: 1340–1355. 38370060 10.1002/fsn3.3847 PMC10867499

[fsn370066-bib-0050] Pahlavanzadeh, H. , A. Basiri , and M. Zarrabi . 2007. “Determination of Parameters and Pretreatment Solution for Grape Drying.” Drying Technology an International Journal 19, no. 1: 217–225.

[fsn370066-bib-0051] Patidar, A. , S. Vishwakarma , and D. Meena . 2021. “Traditional and Recent Development of Pretreatment and Drying Process of Grapes During Raisin Production: A Review of Novel Pretreatment and Drying Methods of Grapes.” Food Frontiers 2, no. 1: 46–61. 10.1002/fft2.64.

[fsn370066-bib-0052] Pawar, D. A. , S. K. Giri , and A. K. Sharma . 2023. “Novel Alternative Pretreatment Approaches for Production of Quality Raisins From Grapes: Opportunities and Future Prospects.” Journal of Food Process Engineering 46, no. 4: e14305. 10.1111/jfpe.14305.

[fsn370066-bib-0053] Re, R. , N. Pellegrini , A. Proteggente , A. Pannala , M. Yang , and C. Rice‐Evans . 1999. “Antioxidant Activity Applying an Improved ABTS Radical Cation Decolorization Assay.” Free Radical Biology and Medicine 26, no. 9: 1231–1237. 10.1016/S0891-5849(98)00315-3. 10381194

[fsn370066-bib-0054] Salengke, S. , and S. K. Sastry . 2005. “Effect of Ohmic Pretreatment on the Drying Rate of Grapes and Adsorption Isotherm of Raisins.” Drying Technology 23: 551–564. 10.1081/DRT-200054131.

[fsn370066-bib-0055] Serio, S. , M. D. Rivero‐Perez , A. C. Correia , A. M. Jordao , and M. L. Gonzalez‐San Jose . 2014. “Analysis of Commercial Grape Raisins: Phenolic Content, Antioxidant Capacity and Radical Scavenger Activity.” Ciência e Técnica Vitivinícola 29, no. 1: 1–8.

[fsn370066-bib-1008] Singleton, V. L. , and J. A. Rossi . 1965. “Colorimetry of Total Phenolics With Phosphomolybdic‐Phosphotungstic Acid Reagents.” American Journal of Enology and Viticulture 16, no. 3: 144–158.

[fsn370066-bib-0056] Söylemezoğlu, G. 2003. “Üzümde Fenolik Bileşikler.” Gida 28, no. 3: 277–285.

[fsn370066-bib-0057] Srivastava, A. , A. Anand , A. Shukla , A. Kumar , D. Buddhi , and A. Sharma . 2021. “A Comprehensive Overview on Solar Grapes Drying: Modeling, Energy, Environmental and Economic Analysis.” Sustainable Energy Technologies and Assessments 47: 101513. 10.1016/j.seta.2021.101513.

[fsn370066-bib-0058] Teker, T. , A. CandemİR , and P. DOĞAN . 2023. “Bağcılıkta Kaolin (Surround WP) Uygulamasının Çekirdeksiz Kuru Üzüm Renk ve Kuruma Randımanı Üzerine Etkisi.” Bahçe 52, no. Özel Sayı 1: 391–395.

[fsn370066-bib-0059] Toor, R. K. , and G. P. Savage . 2006. “Effect of Semi‐Drying on the Antioxidant Components of Tomatoes.” Food Chemistry 94, no. 1: 90–97. 10.1016/j.foodchem.2004.10.054.

[fsn370066-bib-0060] Turgut, S. S. , E. Küçüköner , and E. Karacabey . 2018a. “Improvements in Drying Characteristics and Quality Parameters of Tomato by Carbonic Maceration Pretreatment.” Journal of Food Processing and Preservation 42, no. 2: e13282. 10.1111/jfpp.13282.

[fsn370066-bib-0061] Turgut, S. S. , E. Küçüköner , and E. Karacabey . 2018b. “Influence of Carbonic Maceration Pre‐Treatment on Functional Quality of Dried Tomato Quarters.” Food and Bioprocess Technology 11, no. 10: 1818–1827. 10.1007/s11947-018-2145-y.

[fsn370066-bib-0062] Wang, J. , W.‐S. Mu , X.‐M. Fang , et al. 2017. “Pulsed Vacuum Drying of Thompson Seedless Grape: Effects of Berry Ripeness on Physicochemical Properties and Drying Characteristic.” Food and Bioproducts Processing 106: 117–126. 10.1016/j.fbp.2017.09.003.

[fsn370066-bib-0063] Wang, J. , A. S. Mujumdar , W. Mu , et al. 2016. “Grape Drying: Current Status and Future Trends.” In Grape and Wine Biotechnology, edited by A. Morata , 145–165. IntechOpen.

[fsn370066-bib-0064] Wang, Y. , H. Tao , J. Yang , et al. 2014. “Effect of Carbonic Maceration on Infrared Drying Kinetics and Raisin Qualities of Red Globe (*Vitis vinifera* L.): A New Pre‐Treatment Technology Before Drying.” Innovative Food Science & Emerging Technologies 26: 462–468. 10.1016/j.ifset.2014.09.001.

[fsn370066-bib-0065] Wojdyło, A. , A. Figiel , and J. Oszmiański . 2009. “Effect of Drying Methods With the Application of Vacuum Microwaves on the Bioactive Compounds, Color, and Antioxidant Activity of Strawberry Fruits.” Journal of Agricultural and Food Chemistry 57, no. 4: 1337–1343. 10.1021/jf802507j. 19170638

[fsn370066-bib-0066] Xiao, H. W. , C. L. Pang , L. H. Wang , J. W. Bai , W. X. Yang , and Z. J. Gao . 2010. “Drying Kinetics and Quality of Monukka Seedless Grapes Dried in an Air‐Impingement Jet Dryer.” Biosystems Engineering 105, no. 2: 233–240. 10.1016/j.biosystemseng.2009.11.001.

[fsn370066-bib-0067] Zemni, H. , A. Sghaier , R. Khiari , et al. 2017. “Physicochemical, Phytochemical and Mycological Characteristics of Italia Muscat Raisins Obtained Using Different Pre‐Treatments and Drying Techniques.” Food and Bioprocess Technology 10: 479–490.

[fsn370066-bib-0068] Zhao, D. , Y. Wang , Y. Zhu , and Y. Ni . 2016. “Effect of Carbonic Maceration Pre‐Treatment on the Drying Behavior and Physicochemical Compositions of Sweet Potato Dried With Intermittent or Continuous Microwave.” Drying Technology 34, no. 13: 1604–1612. 10.1080/07373937.2016.1138231.

[fsn370066-bib-0069] Zhong, J. , X. Duan , H. Qu , et al. 2007. “Effects of Various Extraction Conditions on Phenolic Contents and Their Antioxidant Activities of Litchi Fruit Pericarp.” Paper presented at the Europe‐Asia Symposium on Quality Management in Postharvest Systems‐Eurasia 2007. 804.

